# PP23 Machine-Learning-Based Evaluation Of Community Resilience Through Social Media During The First Post-COVID-19 Reopening In China

**DOI:** 10.1017/S026646232400196X

**Published:** 2025-01-07

**Authors:** Shouchuang Zhang, Lanyue Zhang, Jiayi Weng, Yiying Yang, Zefeng Chen, Weiyan Jian

## Abstract

**Introduction:**

The Chinese government lifted most COVID-19 pandemic restrictions in December 2022, triggering a spike in confirmed cases and higher demand for medications. Consequently, a significant number of residents resorted to social media to seek assistance. This study aimed to evaluate community resilience by leveraging Weibo user datasets, coupled with interpretable machine learning (ML)-based techniques, to identify important resilience characteristics.

**Methods:**

Datasets geotagged from the Sina Weibo social media platform between 8 December 2022 and 7 January 2023 were crawled using search terms of “help-seeking” and the keywords of conventional drugs. This study utilized natural language processing (NLP) to label COVID-19-related posts to identify the type of posts, stakeholders’ behaviors, and other information. We built a comprehensive evaluation model, and five ML-based algorithms were compared for analyzing community resilience. Local interpretable model-agnostic explanations (LIME) was employed to verify five models and the XGBoost algorithm showed optimal effects. Shapley Additive Explanations (SHAP) elucidated the best model’s outputs and estimated contributions for key resilience characteristics.

**Results:**

For this study, 199,709 posts were collected. Out of these, 48,425 posts were identified as help-seeking posts, with more than two-thirds receiving responses from community level. The area under curve (AUC) of the XGBoost model was 0.82 (95% confidence interval [CI]: 0.82, 0.83), and the values of accuracy and F1 score were 0.72 and 0.80, respectively. This result demonstrated that the model can successfully evaluate community resilience and subsequently identify the features driving this outcome. Collective efficacy in providing aid, support from official rescue guidelines, and residents’ rapid response to rescue information were identified as the most important characteristics for evaluating community resilience.

**Conclusions:**

This study is the first to harness social media data to quantify community resilience in China based on a framework we developed. Five updated ML-based algorithms were developed to evaluate community resilience, and XGBoost showed optimal effects. Three characteristics of community resilience were found as potential predictors that can enhance decision-making support to reshape health emergency rescue activities.

